# Cardioprotective Effect of Ghrelin in Cardiopulmonary Bypass Involves a Reduction in Inflammatory Response

**DOI:** 10.1371/journal.pone.0055021

**Published:** 2013-01-24

**Authors:** Yukun Cao, Jun Tang, Ting Yang, Heng Ma, Dinghua Yi, Chunhu Gu, Shiqiang Yu

**Affiliations:** 1 Department of Cardiovascular Surgery, Xijing Hospital, Fourth Military Medical University, Xi’an, Shaanxi Province, China; 2 Department of Anesthesiology, School of Stomatology, Fourth Military Medical University, Xi’an, Shaanxi Province, China; 3 Department of Oral Anatomy and Physiology and TMD, School of Stomatology, Fourth Military Medical University, Xi’an, Shaanxi Province, China; University of Pecs Medical School, Hungary

## Abstract

**Background:**

Ghrelin has been reported to protect the cardiovascular system; however, the cardioprotective effect of ghrelin against cardiopulmonary bypass (CPB) induced myocardial injury are unclear. In this study, the protective effect of ghrelin on CPB induced myocardial injury and the underlying mechanisms were investigated.

**Methods and Results:**

Adult male rats were subjected to CPB and randomly to receive vehicle (n = 8), ghrelin (n = 8), ghrelin plus [D-Lys3]-GHRP-6, a GHSR-1a inhibitor (n = 8), or ghrelin plus wortmannin, a phosphoinositide 3′-kinase (PI3K) inhibitor (n = 8). *In vitro* study was performed on cultured cardiomyocytes subjected to simulated cardiopulmonary bypass (SCPB). Ghrelin attenuated the inﬂammatory response, as evidenced by reduced induction of TNF-α, IL-6 and myocardial myeloperoxidase activity and concurrent reduction in apoptosis, oxidative stress, and levels of myocardial injury markers following CPB. Moreover, ghrelin significantly increased cardiac function after CPB. In cultured cardiomyocytes subjected to simulated CPB, ghrelin increased cell viability and decreased the percentage of apoptotic myocytes. Inhibition of ghrelin downstream signaling blocked the cardioprotective effects both *in vivo* and *vitro*.

**Conclusions:**

Ghrelin could provide an effective approach to the attenuation of CPB induced myocardial injury. The cardioprotective effects elicited by ghrelin may contribute to the inhibition of inflammatory response through the Akt-activated pathway.

## Introduction

Open heart surgery with cardiopulmonary bypass (CPB) is associated with both acute systemic and myocardial inﬂammatory responses, which have implications for postoperative myocardial function and recovery [1]. In addition, the heart itself is a source of inflammatory mediators and reactive oxygen species that are likely to contribute to the impairment of cardiac pump function [2]. Despite significant changes and improvements in surgical techniques, inflammation remains a significant clinical problem. Therefore, the complementary and alternative medicine to control the inflammatory response continues to be the focus of extensive experimental research and clinical studies [3].

Ghrelin is a 28-amino-acid acylated peptide, produced primarily by the X/A-like enteroendocrine cells of the stomach that has been identified as the endogenous ligand for the growth hormone secretagogue receptor (GHSR) [4]. Within cardiovascular system, two GHS-R subtypes, GHS-R1a and GHS-R1b, are present in the heart and in the vessels [5], indicating a potential role of ghrelin in regulating cardiovascular function. Stimulation of GHSR prevents cardiac damage after ischemia/reperfusion in hypophysectomized rats [6]. Moreover, administration of ghrelin improved cardiac function and reduced myocardial injury induced by ischemia and reperfusion in the isolated rat heart, effects that were independent of growth hormone secretion and have been related to protein kinase C activation [7], [8]. Ghrelin attenuates the inflammatory response in endotoxic shock [9], [10], stroke [11], [12], and acute myocardial infarction [13]. In particular, ghrelin has been shown to alleviate LV dysfunction and ventricular remodeling post-MI in the rat, by inhibiting the inflammatory response [14], [15]. These observations have indicated that ghrelin functions as an anti-inﬂammatory molecule, although the underlying mechanisms remain elusive.

Tumor necrosis factor (TNF)-α and interleukin (IL)-6 are both important and potent pro-inﬂammatory cytokines. TNF-α is an important pro-inﬂammatory factor, which is able to activate other inﬂammatory cytokines and induce the expression of adhesion molecules. IL-6 is responsible for the coordination of the acute phase response and plays a positive role in the local inﬂammatory reaction by amplifying leukocyte accumulation [16], [17]. Elevated levels of serum TNF-α and IL-6 have been reported in patients with ischemia/reperfusion injury, chronic heart failure, and viral myocarditis [18], [19], [20]. However, whether ghrelin decreases inﬂammatory response, especially TNF-α and IL-6 induction, in CPB induced myocardial inflammatory injury, has not been previously explored. Inﬂammatory response and cytokine production are particularly active after CPB and contribute to the development of cardiac dysfunction [21]. Cytokines such as TNF-α and IL-6 released after CPB induced myocardial inflammatory injury can acutely regulate myocyte survival or death and trigger subsequent cellular inﬂammatory response. Moreover, both TNF-α and IL-6 exert direct negative inotropic effects on the heart, that can contribute to myocardial dysfunction in several pathological conditions [22], [23]. In the present study, accordingly, we investigated whether ghrelin attenuates myocardial injury induced by cardiopulmonary bypass and explored the mechanisms underlying this protection.

## Materials and Methods

### Animals and Cardiopulmonary Bypass Model

The experiments were performed in adherence with the National Institutes of Health Guidelines for the Use of Laboratory Animals and were approved by the Fourth Military Medical University Committee on Animal Care. Male Sprague–Dawley rats (14 weeks old, 450–550 g) were used for the experiments (n = 8/group). The rat cardiopulmonary bypass model was developed according to Dong and colleagues with some modifications [24], [25]. Rats were anesthetized with butaylone (60 mg/kg, intraperitoneal administration) at the beginning, and additional butaylone was used to maintain anesthesia. The right femoral artery was cannulated for arterial pressure monitoring. After administration of heparin (250 U/kg), a 16 gauge catheter, modified to a multiside-orifices cannula in the forepart, was inserted into the right jugular vein and advanced to the right atrium. This approach was used as a vein drain line in CPB. A 22 gauge catheter for arterial infusion was introduced into tail artery. The mini-cardiopulmonary bypass circuit comprised a venous reservoir (10 ml), a specially designed membrane oxygenator, and a roller pump (BT00-300M, Lange Co, Shanghai, China). All components were connected with polyethylene tubing. Body central temperature was monitored with a rectal probe and kept at 36.5°C to 38.3°C by a heat lamp placed around the animal and the CPB equipment. The circuit was primed with 10 mL of a solution of heparin 1 ml (250 U/kg), synthetic colloid 8 ml and sodium bicarbonate 1 ml. At the initiation of perfusion, the ﬂow rate was gradually increased to 100 ml/kg/min and maintained for 60 min; it was then turned down step by step to maintain hemodynamic stability. As the rat was weaned from CPB, the tail artery catheter was removed, and the right jugular vein catheter was drawn back to the superior vena. The remaining priming solution was infused gradually when the main arterial pressure was less than 60 mm Hg. After 1 h of intensive postoperative care, the right jugular vein catheter and the femoral artery catheter were decannulated. Then the neck, tail, and groin incisions were sutured. Throughout the experiment, the mean arterial pressure was maintained about 60 to 80 mm Hg.

Before the start of CPB, rats randomly received one of the following treatments: Sham group (only cannulated for cardiopulmonary bypass but not undergoing cardiopulmonary bypass); Control group (intravenous injection; saline); Ghrelin group (intravenous infusion at 60 µg/kg per h ghrelin from 10 min before the start of CPB to the end of the experiment, Phoenix Pharmaceuticals, Burlingame, CA, USA); ghrelin plus [D-Lys3]-GHRP-6 (a GHSR-1a blocker, Phoenix, Pharmaceuticals, USA) group (40 µg/kg intravenous injection 15 min before CPB); ghrelin plus wortmannin (a PI3K inhibitor, Sigma, USA) group (20 µg/kg intravenous injection 15 min before CPB). After 4 h of cardiopulmonary bypass, the rats were anesthetized with butaylone again. A micro-catheter was inserted into left ventricular through left carotid artery to measure the left ventricular developed pressure (LVDP). The artery pressure was measured by right femoral artery cannulation. Electrocardiogram (ECG) and LVDP were simultaneously recorded on a polygraph (RM-6200C, Chengdu, China). Mean arterial blood pressure (MAP), heart rate (HR), left ventricular developed pressure (LVDP), and the instantaneous first derivation of LVP (±LVdP/dt_max_) were derived by computer algorithms. After these operations for function measurement, blood samples were collected by cardiac puncturing and the heart was harvested for biochemical analysis. Plasma lactate dehydrogenase (LDH) levels were measured spectrophotometrically (DU 640; Beckman Coulter, Brea, CA). And plasma cardiac troponin I (cTnI) levels were measured by a commercially available high-sensitivity cTnI ELISA kit (Wuhao Company, Shanghai, China). All measurements were performed in duplicates.

### Determination of Serum and Tissue Levels of Inﬂammatory Cytokines and Myocardial Myeloperoxidase Activity

After 4 h of CPB, the levels of TNF-α and IL-6 in the myocardial tissue homogenates and serum were determined with commercially available enzyme-linked immunosorbent assay (ELISA) kits (R&D Systems). All ELISA protocols were carried out according to manufacturer guidelines as described in our previous study [21]. Myeloperoxidase (MPO) activity was determined as described previously [26].

### Reduced Glutathione/Oxidized Glutathione Analysis

Hearts (n = 8 per group) were rapidly excised and the left ventricles were frozen in liquid nitrogen for the glutathione assay. Samples were homogenized with a glass tissue grinder. Colorimetric determination of reduced (GSH) and oxidized (GSSG) glutathione content was performed using a commercial kit (Beyotime Institute of Biotechnology, Shanghai, China).

### Cardiomyocyte Isolation and Contractions

Cardiomyocytes were enzymatically isolated from rats following CPB as described previously [27]. Myocyte yield was approximately 75%. Only rod-shaped cells with clear edges were selected for contractile study. Contractile properties of myocytes were assessed using an IonOptixTM soft-edge system [27]. Myocyte contraction was induced at a frequency of 0.5 Hz by platinum electrodes connected to an electrical stimulator. Cell shortening and relaxation were assessed using the following indices: peak shortening (PS), time-to-PS (TPS), time-to-90% relaxation (TR90), and maximal velocities of shortening/relaxation (±dL/dt).

### Terminal Deoxynucleotidyl Nick-end Labeling Assay and Myocardial Caspase-3 Activity

Myocardial apoptotic index was analyzed by terminal deoxynucleotidyl nick-end labeling (TUNEL) assay as documented in a previous study [28]. The index of apoptosis was expressed by number of apoptotic myocytes/total number ×100%. And myocardial caspase-3 activity was performed by using a caspase-3 colorimetric assay kit (Chemicon, Temecula, CA) following the manufacturer’s instructions as previously documented [29]. The activity of caspase-3 was calculated using a standard curve and expressed as fold increase over the mean value of the sham CPB group.

### Simulated Cardiopulmonary Bypass (SCPB)

Primary cultures of neonatal rat cardiomyocytes from one to two day old Sprague-Dawley rats were prepared and cultured as described previously [30]. SCPB was performed as described by Aebert with some modifications [31]. Briefly, the cardiomyocytes were transferred into the CPB buffer containing 20% CPB serum (taken from rats undergoing CPB) and incubated for 6 h in a humidified cell culture incubator (5% CO_2_, 37°C). This buffer was designed to simulate the extracellular milieu of CPB, with the approximate concentrations of potassium, hydrogen, and lactate ions that occur *in vivo*. At the onset of SCPB culture, cardiomyocytes (n = 8/group) were randomly exposed to one of the following treatments: vehicle, ghrelin (10 nmol/L), ghrelin plus [D-Lys3]-GHRP-6 (15 nmol/L), and ghrelin plus wortmannin (10 nmol/L). Cell viability was determined with the MTT assay. After SCPB, myocyte apoptosis was determined by means of DAPI/TUNEL double-staining procedure. Among all the DAPI positive stained cardiomyocyte, the number of TUNEL-positive cells was presented as a percentage. Neither [D-Lys3]-GHRP-6 nor wortmannin treatment alone significantly affected cell viability (p = 0.25 and 0.34, respectively).

### Immunoblotting

Total protein was extracted from both CPB cardiac tissue and SCPB myocytes, and the immunoblots were probed with an anti-pAkt (Ser-473) antibody (Cell Signaling, Santa Cruz, CA) overnight at 4°C followed by incubation with the corresponding secondary antibody at room temperature for 1 h. The blots were visualized with ECL-Plus reagent (GE Healthcare, Piscataway, NJ). pAkt immunoblots was then stripped with strip buffer at 50°C for 30 min and re-blotted for total Akt.

### Statistical Analysis

All values were expressed as mean±SEM. Data were analyzed using a commercially available statistics software package (GraphPad Prism software, version 5.0,GraphPad Software, San Diego, CA, USA). For repeatedly measured parameters, comparisons between the groups were analyzed by two-way ANOVA. Otherwise, one-way analysis of variance (ANOVA) followed by Tukey’s test for post hoc analysis was adopted for comparisons. A *P<*0.05 was considered as statistically significant.

## Results

### Ghrelin Decreased Myocardial Injury and Oxidative Stress in CPB Rats

**Figure 1:**
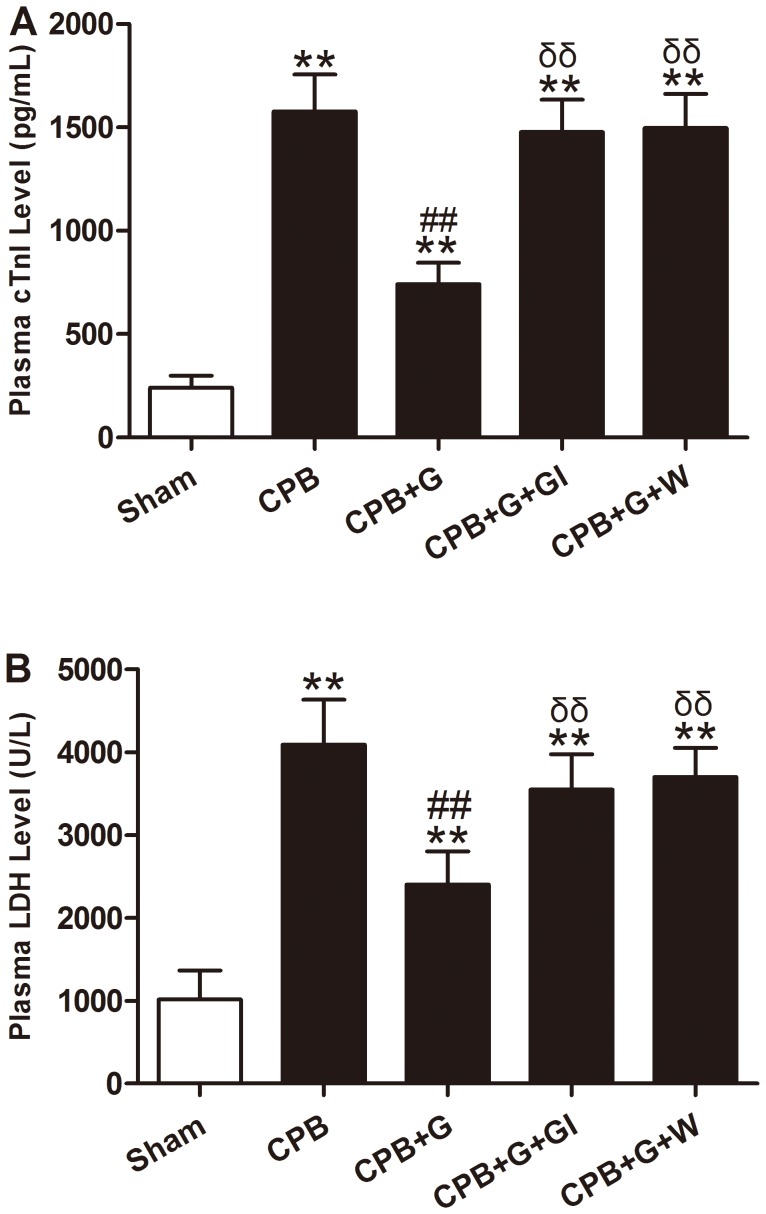
Myocardial injury in rats subjected to cardiopulmonary bypass (CPB) with different treatments (n = 8/group). Values presented are mean±SEM. A: serum cardiac troponin I (cTnI) level. B: serum lactate dehydrogenase (LDH) level. G, ghrelin; GI, [D-Lys3]-GHRP-6, ghrelin inhibitor; W, wortmannin, a PI3K inhibitor. **P<0.01 vs. Sham, ^##^ *P<*0.01 vs. CPB, ^δδ^ *P<*0.01 vs. CPB+G.

**Figure 2:**
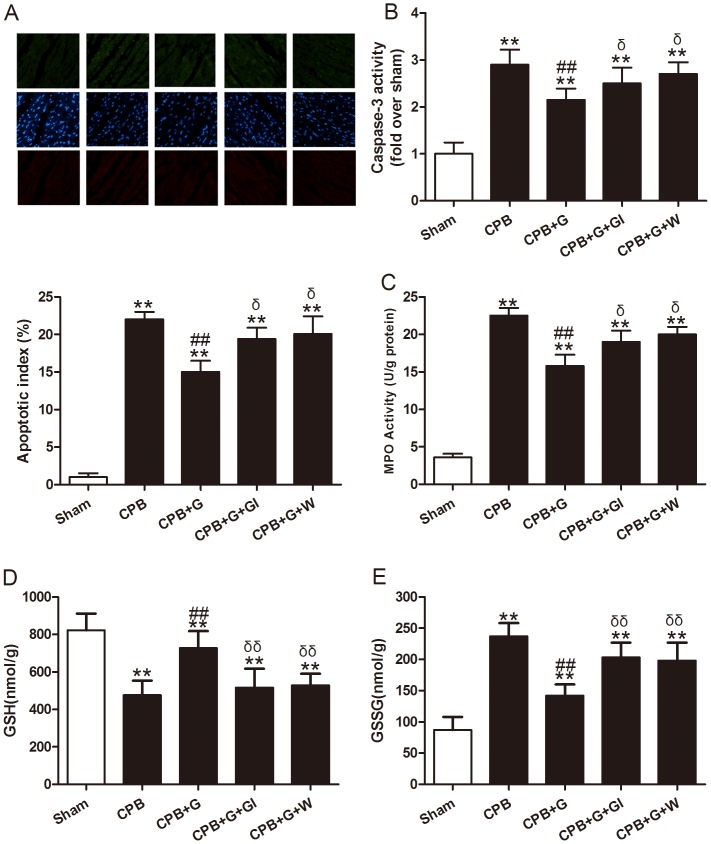
Myocardial apoptosis, MPO activity, reduced glutathione (GSH) and oxidized glutathione (GSSH) in rats subjected to cardiopulmonary bypass (CPB) with different treatments (n = 8/group). A, top: representative photomicrographs of in situ detection of apoptotic myocytes by terminal deoxynucleotidyl nick-end labeling (TUNEL) staining in ischemic heart tissue from rats subjected to 60 min CPB. Green ﬂuorescence shows TUNEL-positive nuclei; blue ﬂuorescence shows nuclei of total cardiomyocytes. Bottom: percentage of TUNEL-positive nuclei in heart tissue sections. (x20 objective) B: myocardial caspase-3 activity. C: MPO activity. D: reduced glutathione (GSH). E: oxidized glutathione (GSSH). Values presented are mean±SEM. G, ghrelin; GI, [D-Lys3]-GHRP-6, ghrelin inhibitor; W, wortmannin, a PI3K inhibitor. **P<0.01 vs. Sham, ^##^ *P*<0.01 vs. CPB, ^δ^ *P*<0.05 vs. CPB+G.

To examine whether ghrelin might reduce myocardial injury, plasma cTnI and LDH levels, myocardial apoptosis and caspase-3 activity were measured. As shown in Figs. 1 and 2, 60 min of CPB resulted in myocardial injury, as evidenced by increased serum cTnI and LDH activity (Fig. 1), myocardial apoptosis (Fig. 2A), and caspase-3 activity (Fig. 2B). Ghrelin treatment significantly reduced cTnI and LDH activity, myocardial apoptosis, and caspase-3 activity compared with the vehicle group (all *P*<0.05).

Because CPB elicits a robust neutrophil response, we determined whether ghrelin exerted its cardioprotective effect by an anti-neutrophil mechanism. Neutrophil accumulation was determined in the CPB hearts by measuring MPO activity. As illustrated in Fig. 2C, 60 min of CPB significantly elevated MPO activity. Ghrelin treatment significantly reduced CPB-induced elevation in MPO activity, indicating a significant anti-neutrophil effect of ghrelin. Reduced (GSH) and oxidized (GSSG) glutathione content are considered good indicators of oxidant status. We investigated whether ghrelin was able to counteract oxidative stress in this model by detecting GSH/GSSG. GSH levels were significantly lower and GSSG significantly higher in CPB rats compared with sham-operated rats. This effect was completely reverted by ghrelin treatment, indicating that ghrelin could attenuate oxidative stress induced by CPB (Fig. 2 D and E, all *P*<0.05). These effects of ghrelin were abolished by [D-Lys3]-GHRP-6, a GHSR-1a inhibitor and Wortmannin, a specific PI3K inhibitor.

### Ghrelin Preserved Cardiac Pump Function after CPB

**Figure 3:**
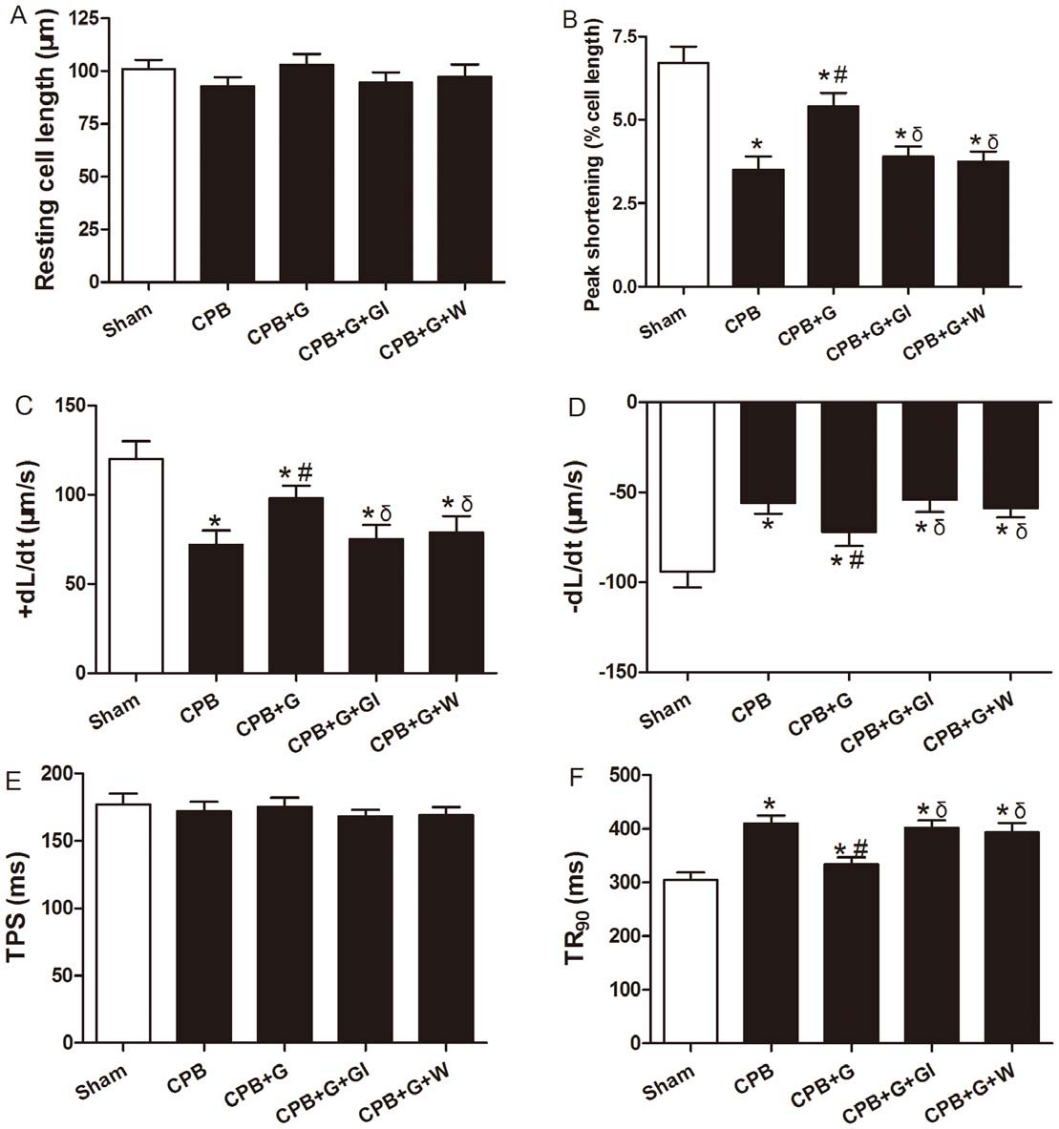
Cardiomyocyte contractile properties following CPB. (A) Resting cell length; (B) peak shortening (PS, normalized to cell length); (C) maximal velocity of shortening (+dL/dt); (D) maximal velocity of relaxtion (-dL/dt); (E) time-to-PS (TPS) and (F) time-to-90% relaxtion (TR90). Values presented are mean±SEM, n = 68–74 cells for each group from four rats per group. G, ghrelin; GI, [D-Lys3]-GHRP-6, ghrelin inhibitor; W, wortmannin, a PI3K inhibitor. *P<0.05 vs. Sham, ^#^ *P<*0.05 vs. CPB, ^δ^ *P<*0.05 vs. CPB+G.

As shown in Table 1, CPB decreased MAP, LVDP, +LV dP/dt_max_ and −LV dP/dt_max_ compared with the sham group. Treatment with ghrelin improved these parameters of cardiac function compared with the CPB group and these beneficial effects were abolished with the ghrelin inhibitor and PI3K inhibitor (P<0.05). There was no significant difference in the changes of HR between groups treated with either ghrelin or saline. These results showed that treatment with ghrelin improves cardiac systolic and diastolic function in rats subjected to CPB. We went on to examine the effect of ghrelin on CPB-elicited cardiomyocyte contractile response. CPB triggered an overt contractile dysfunction manifested as depressed peak shortening (PS) and maximal velocity of shortening/relaxation (±dL/dt), as well as prolonged TR90 in vehicle treated cardiomyocytes. In line with its protective effects on myocardial injury, ghrelin significantly alleviated CPB-induced cardiomyocyte contractile dysfunction. The effect of ghrelin was eliminated by [D-Lys3]-GHRP-6 and Wortmanin. (Fig. 3).

**Table 1:** Effects of ghrelin on cardiac function in different groups after CPB (n = 8, mean±SEM).

Group	HR (beats/min)	MAP (mmHg)	LVDP (mmHg)	+LVdp/dt_max_ (mmHg/s)	−LVdp/dt_max_ (mHg/s)
Sham	331±10	97±9	101±12	3211±157	2864±184
CPB	327±15	61±7*	68±8*	2654±117*	2357±147*
CPB+G	335±11	81±8* #	89±11* #	2907±168* #	2629±142* #
CPB+G+GI	329±17	68±5* δ	74±9* δ	2743±134* δ	2417±162* δ
CPB+G+W	328±13	65±7* δ	72±10* δ	2711±139* δ	2397±154* δ

G, ghrelin; GI, [D-Lys3]-GHRP-6, ghrelin inhibitor; W, wortmannin, a PI3K inhibitor; HR, heart rate; MAP, mean arterial blood pressure; LVDP, left ventricular developed pressure; ±LVdP/dt_max_, the instantaneous first derivation of left ventricle pressure.

* *P*<0.05 vs. Sham,

# *P*<0.05 vs. CPB,

δ *P*<0.05 vs. CPB+G.

### Ghrelin Inhibited Systemic and Myocardial Inﬂammatory Response in CPB Rats

**Figure 4:**
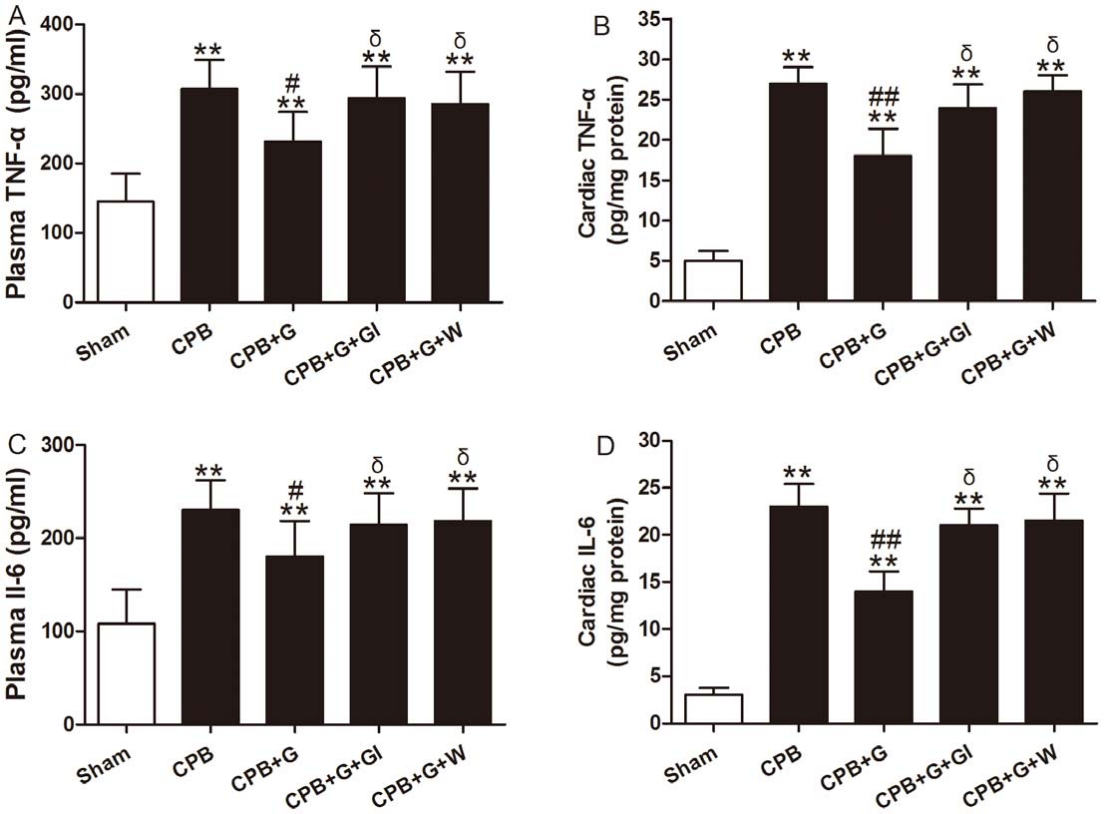
Serum (A) and cardiac (B) tumor necrosis factor (TNF)-α and Serum (C) and cardiac (D) interleukin (IL)-6 production in rats subjected to CPB with different treatments (n = 8/group). Values presented are mean±SEM. G, ghrelin; GI, [D-Lys3]-GHRP-6, ghrelin inhibitor; W, wortmannin, a PI3K inhibitor. **P<0.01 vs. Sham, ^##^ *P<*0.01 vs. CPB, ^#^ *P<*0.05 vs. CPB, ^δ^ *P<*0.05 vs. CPB+G.

Previous studies demonstrated that TNF-αand IL-6 are multifunctional pro-inﬂammatory cytokines regulating neutrophil infiltration [16], [17]. Therefore, we examined serum and cardiac TNF-α and IL-6 concentrations. Before the initiation of CPB, serum IL-6 and TNF-α were not detectable in any of the groups. Serum TNF-α and IL-6 levels were increased significantly in the vehicle CPB group, and ghrelin treatment attenuated this increase (*P<*0.01 for both, Fig. 4A and C). In myocardial extracts, CPB increased the production of TNF-α and IL-6 significantly. Compared with vehicle CPB group, ghrelin treatment significantly decreased tissue levels of TNF-α and IL-6 (Fig. 4B and D; both *P<*0.01). The effect of ghrelin was abolished by [D-Lys3]-GHRP-6 and Wortmanin. These data suggest that ghrelin reduces the systemic and myocardial inﬂammatory responses in CPB.

### Ghrelin Decreased Cardiomyocyte Injury Following Simulated CPB

**Figure 5:**
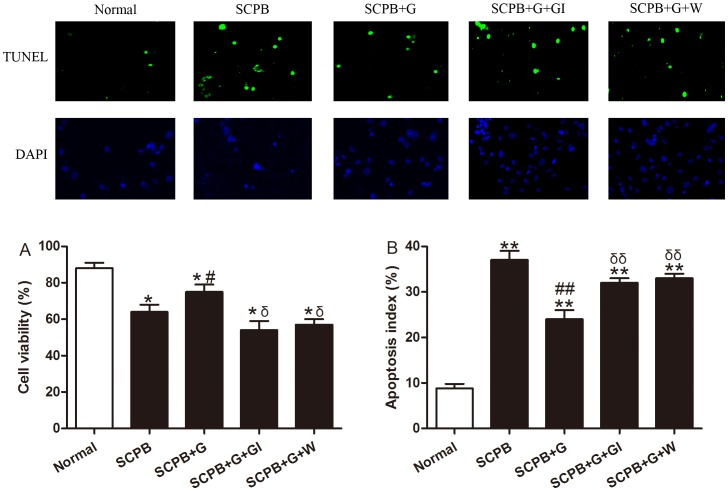
Cell viability and apoptosis of simulated CPB cardiomyocytes following different treatments. A, cell viability measured by MTT assay (n = 8). B, apoptosis measured by in situ detection of TUNEL-positive nuclei and apoptotic index expressed as the percentage of TUNEL-positive myocytes (top row) over total nuclei determined by 4′,6-diamino-2-phenylindole staining (bottom row) (×20 objective). (n = 8). Values presented are mean±SEM. Normal, normal culture condition; SCPB, simulated CPB; G, ghrelin; GI, [D-Lys3]-GHRP-6, ghrelin inhibitor; W, wortmannin, a PI3K inhibitor. **P<0.01 vs. Sham, *P<0.05 vs. Sham, ^##^ *P<*0.01 vs. CPB, ^#^ *P<*0.05 vs. CPB, ^δδ^ *P<*0.01 vs. CPB+G, ^δ^ *P<*0.05 vs. CPB+G.

Our *in vivo* evidence indicated that ghrelin may exert its cardioprotective effect via a novel anti-inﬂammatory mechanism. To determine if there was also a direct effect on myocyte function, we cultured neonatal cardiomyocytes and subjected the cells to 6 h of SCPB. As shown in Fig. 5A, SCPB decreased cell viability compared to normal culture conditions (*P<*0.05) and ghrelin increased cell viability (*P<*0.05). Apoptosis is the major form of myocyte cell death after CPB. To investigate whether ghrelin decreased myocardial apoptosis after SCPB, the apoptotic myocytes/total number in cultured cardiomyocytes were determined. In cardiomyocytes from normal culture conditions, there was a very low rate of apoptosis. In contrast, a significant number of apoptotic cells were observed in cardiomyocytes subjected to SCPB. Treatment of ghrelin shortly before SCPB reduced the percentage of apoptotic myocytes (Fig. 5B; *P<*0.01). The effect of ghrelin was blocked by [D-Lys3]-GHRP-6 and Wortmanin.

### Activation of GHSR-1a Activated Akt Through Phosphatidylinositol 3′-kinase (PI3K)

**Figure 6:**
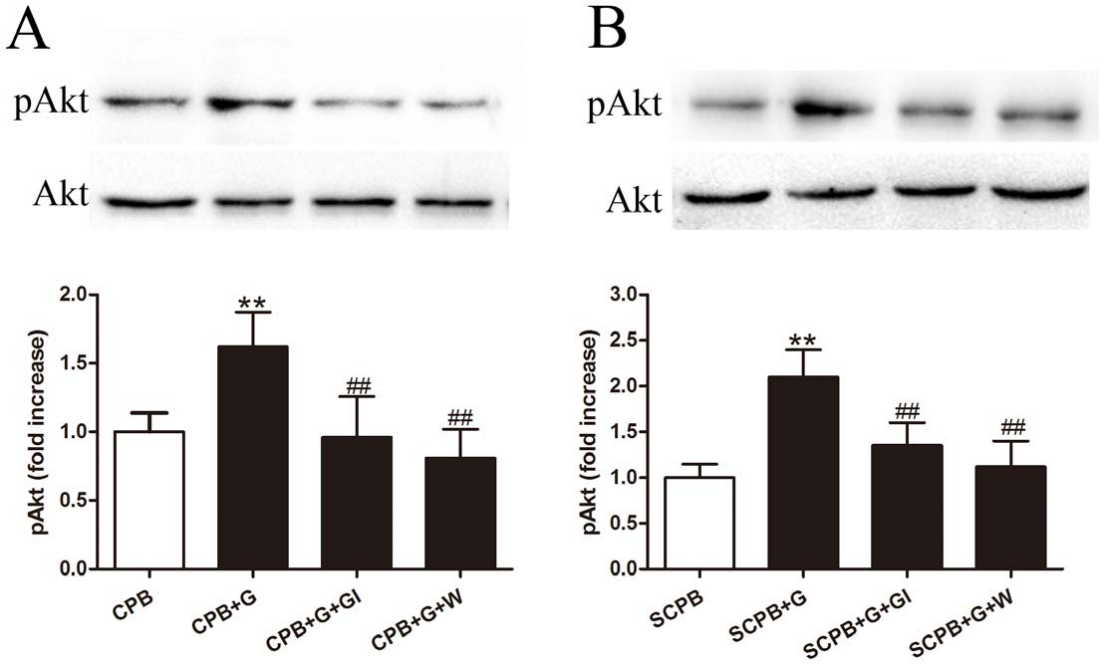
Phosphorylation of Akt *in vivo* (A) and *in vitro* (B) following different treatments. Values presented are mean±SEM. N = 6; G, ghrelin; GI, [D-Lys3]-GHRP-6, ghrelin inhibitor; W, wortmannin, a PI3K inhibitor. **P<0.01 vs. Sham, ^##^ *P<*0.01 vs. CPB or SCPB.

Ghrelin treatment resulted in increased Akt phosphorylation both *in vivo* and *in vitro* (Fig.6). To further investigate the mechanisms underlying the ghrelin-induced anti-inﬂammatory effects, we measured Akt expression and Akt phosphorylation by immunoblotting of myocardial protein extracts from rats at the end of the *in vivo* experiment and from protein extracts from cultured cardiomyocytes exposed to SCPB (Fig. 6). There was no significant difference in total Akt levels among the different groups, whereas treatment with ghrelin significantly increased the phosphorylation of Akt (*P<*0.01). Pretreatment with [D-Lys3]-GHRP-6 and wortmannin completely blocked the Akt phosphorylation induced by ghrelin (both *P<*0.01). These results demonstrated that both *in vivo* and *in vitro* treatment with ghrelin at the onset of CPB increased Akt phosphorylation and Akt activity through the activation of both GHSR-1a and PI3-kinase pathways.

## Discussion

Several important observations were made from the present study. First, we provided direct *in vivo* evidence that ghrelin inhibited systemic and local inflammatory responses in CPB rats. Second, we demonstrated for the first time that *in vivo* treatment with ghrelin attenuated myocardial injury by reducing CPB-induced apoptosis, decreasing oxidative stress and myocardial injury markers, and preserving cardiac pump function. Third, we have also demonstrated that *in vitro* treatment with ghrelin exerted cardioprotective effect on cultured myocytes exposed to a simulated cardiopulmonary bypass environment. Finally, we have shown that ghrelin protected the heart undergoing cardiopulmonary bypass against systemic and local inflammatory responses via activation of GHSR-1a and Akt.

Inﬂammatory responses, including cytokine induction and neutrophil infiltration, play a critical role in CPB-induced myocardial injury. Several anti-inﬂammatory procedures and pharmacological agents have been used in cardiac surgery to attenuate myocardial inﬂammatory injury, including the use of leukocyte filtration, corticosteroids, aprotinin, heparin, and nitrogen monoxidum donor compounds [32]. Despite the relatively small number of studies investigating the effects of reducing the inﬂammatory response on myocardial injury, the vast majority of these studies have shown that therapies targeting myocardial inﬂammatory response are beneficial in salvaging myocardium. A prospective observational study has shown that IL-6 and TNF-α should be explored as predicting factors of cardiac dysfunction after cardiovascular surgery with cardiopulmonary bypass [33]. Clinical investigation showed that plasma levels of IL-6 positively associated with the severity of the inﬂammatory response due to cardiopulmonary bypass and also with postoperative morbidity in adults [34]. We showed in this study that serum and myocardial TNF-α and IL-6 levels increase following CPB, and that ghrelin treatment significantly reduced levels of both. Dixi et al. reported that ghrelin suppresses pro-inﬂammatory factors, including TNF-α, and reactive oxygen species generation human monocytes and T cells [35]. Along with our data, ghrelin likely exerts an inhibitory effect on multiple immune cell types. Based on these observations, immune cells are affected by ghrelin treatment in CPB rats. Likewise, MPO activity was elevated, consistent with neutrophil accumulation, in myocardial tissue following CPB. Ghrelin treatment also reduced MPO activity. Taken together, ghrelin suppressed the inﬂammatory response to CPB. Furthermore, ghrelin trearment increased GSH levels and decreased GSSG levels, indicating that ghrelin could counteract oxidative stress induced by CPB. These effects may explain the improved cardiac pump function, as ghrelin significantly increased MAP, LVDP, +LV dP/dt_max_ and -LV dP/dt_max_ *in vivo* and cardiomyocyte contractile function compared *in vitro* with the CPB group.

Our data showed that ghrelin given at the onset of CPB exerted an overall beneficial effect. Open-heart surgery triggers an inflammatory response that is largely the result of global ischaemia during CPB followed by reperfusion injury that occurs following CPB. The heart sustains injury triggered by ischaemia and reperfusion as a result of the effects of systemic inflammatory mediators [2]. Several studies have demonstrated that ghrelin could protect the myocardium against ischemia/reperfusion injury and improve post-myocardium infarction prognosis [8], [35]. *In vitro* treatment with ghrelin significantly decreased apoptosis in cardiomyocytes exposed to simulated CPB, which strongly suggests that ghrelin exerts also cardioprotective effects through direct effects on cardiomyocyte viability.

Previous studies have indicated that the PI3K/Akt pathway serves as a negative regulator for inﬂammatory genes in monocytes [36], macrophages [37], and endothelial cells [38]. In our study, we found that both *in vivo* and *in vitro* treatment with ghrelin significantly increased phosphorylation of Akt. The functional involvement of PI3K/Akt signaling pathways in the cardioprotective effects of ghrelin were demonstrated by the PI3K inhibitor wortmannin. These results suggest that PI3K/Akt pathway is an independent signaling pathway downstream of ghrelin. To our knowledge, the present study is the first to demonstrate that *in vitro* and *in vivo* treatment with ghrelin exerts cardioprotective effects against cardiomyocyte injury by stimulating the PI3K/Akt pathway.

GHSR is a seven-transmembrane G protein-coupled receptor for ghrelin, and stimulation of GHSR with ghrelin leads to activation of G protein, calcium mobilization, and multiple downstream signaling. Many biological actions of ghrelin are initiated by binding of ghrelin to its cognate cell surface receptor GHSR-1a [4], [39], [40]. The GHSR-1a inhibitor [D-Lys3]-GHRP-6 efficiently blocked ghrelin-induced phosphorylation of Akt and in vitro cardioprotective effects, suggesting that ghrelin exerts effects through a receptor linked to GHSR-1a. These results suggest that PI3K/Akt signaling pathway is GHSR-1a-mediated involved in the cardioprotective effects of ghrelin.

Recent data suggest a relevant role of peptides encoded by the ghrelin gene in protecting heart from ischemia, inflammation and oxidation. Obestatin, another peptide mediator deriving together with ghrelin from pre-proGhrelin, exerts remarkable protective effects against myocardial injury, apoptotic cell death and contractile dysfunction induced by ischemia and reperfusion in the heart. Besides to the PI3K/Akt pathway activation, the protective effect of obestatin has been related to stimulation of other reperfusion injury salvage kinases (RISK), such as PKC and ERK 1/2 [41]. Circulating levels of ghrelin or proghrelin-derived peptides are altered in cardiovascular diseases such as cardiac ischemia, type 2 diabetes mellitus and obesity [42], [43], [44]. All of above suggest the critical role of ghrelin in myocardial protection both under physiological and pathological conditions.

In conclusion, we have demonstrated that ghrelin inhibits CPB-induced systemic and local inflammatory mediator production through the GHSR-1a activated and PI3K/Akt-dependent pathway, which results in cardioprotection in response to CPB. The anti-inﬂammatory property elicited by ghrelin, together with its metabolic modulation, anti-apoptosis, anti-oxidant, and vasculoprotection mechanisms [15], implicate ghrelin as a versatile and effective drug not only in CPB but also in other inflammatory diseases, including stroke, sepsis, and shock [9], [10].
